# Assessment of functional health literacy in Brazilian carers of older people

**DOI:** 10.1590/1980-57642018dn13-020006

**Published:** 2019

**Authors:** Kaoana Maria Vieira de Almeida, Christine Toye, Liciana Vaz de Arruda Silveira, Susan Slatyer, Keith Hill, Alessandro Ferrari Jacinto

**Affiliations:** 1 Universidade Estadual Paulista Julio de Mesquita Filho, Faculdade de Medicina Campus de Botucatu Ringgold standard institution, Botucatu, SP, Brazil.; 2 Curtin University Ringgold standard institution, Perth, Australia.

**Keywords:** educational status, health education, health literacy, aged, caregivers, escolaridade, educação em saúde, alfabetização em saúde, idoso, cuidadores

## Abstract

Individuals with low health literacy have less knowledge of their own health condition. Carers play a key role in older people’s activities of daily living.

**Objective:**

to evaluate the performance of carers of older people using the S-TOFHLA (Short Test of Functional Health Literacy in Adults) and to identify caregiver characteristics associated with low functional health literacy.

**Methods:**

a cross-sectional study was conducted. The S-TOFHLA, a sociodemographic instrument, the Mini-Mental State Exam and the Patient Health Questionnaire-2 were applied to 80 carers of older patients routinely followed by doctors from the Primary Health Care Sector of the City of Botucatu, São Paulo. The multivariate analysis used an ordinal logistic regression model with test (S-TOFHLA) scores as the dependent variable. The level of statistical significance adopted was 0.05.

**Results:**

the individuals had a mean age of 54.6 (± 11.7); 27% of the carers evaluated had inadequate levels of health literacy (S-TOFHLA ≥54). A higher proportion of individuals with low education had inadequate or marginal health literacy (p<0.001).

**Conclusion:**

nearly 1/3 of the carers had marginal or inadequate levels of health literacy. These results highlight the difficulties of many carers in understanding health information.

## RESUMO.

Indivíduos com baixo alfabetismo em saúde têm menos conhecimento sobre sua própria condição de saúde. Cuidadores desempenham um papel fundamental nas atividades de vida diária dos idosos.

**Objetivo:**

avaliar o desempenho de cuidadores de pessoas idosas usando o “S-TOFHLA *(Short Test of Functional Health Literacy in Adults)*” e identificar características do cuidador associadas ao baixa alfabetismo em saúde.

**Métodos:**

um estudo transversal foi conduzido. O S-TOFHLA, um instrumento sociodemográfico, o Miniexame do Estado Mental e o *“Patient Health Questionnaire - 2”* foram aplicados a 80 cuidadores de pacientes idosos rotineiramente acompanhados por médicos do Setor de Atenção Primária à Saúde da cidade de Botucatu, São Paulo. A análise multivariada utilizou um modelo de regressão logística ordinal com os escores do S-TOFHLA como variável dependente. O nível de significância estatística adotado foi de 0,05.

**Resultados:**

os indivíduos tiveram idade média de 54,6 (± 11,7) anos; 27% dos cuidadores avaliados apresentavam níveis inadequados de alfabetização em saúde. Uma proporção maior de indivíduos com baixa escolaridade apresentou alfabetização em saúde inadequada e limítrofe (p<0,001).

**Conclusão:**

aproximadamente 1/3 dos cuidadores apresentaram níveis limítrofes e inadequados de alfabetização em saúde. Esses resultados destacam as dificuldades de muitos cuidadores em compreender informações sobre saúde.

lliteracy is a global problem.^1^ Illiterate individuals are defined as those who cannot read or write. There are 774 million people classified as illiterate worldwide and 72% of these are found in 10 countries, including Brazil.^2^

In the healthcare context, low levels of literacy can impede a person’s ability to manage their own health and to access services.^3^^,^ 4

In the absence of mastery of reading and comprehension skills, the individual´s health may be adversely impacted due to a lack of understanding of their health status and medical advice, difficulty interpreting labels and medication instructions, and consequent worse management of self-care activities.^5^^,^ 6 Among older people, low health literacy has been associated with poor health behaviours,^7^ worse health outcomes, and higher mortality rates.^6^ Additionally, a recent study of older people with long-term health conditions (n = 4278) linked low health literacy to poorer physical, psychological, social relationships and environmental quality of life.^8^

With advancing age, the healthcare required by older people increases as comorbidities develop, precipitating declines in physical and functional capacity and the need for more medications. Therefore, many older people need the support of carers to promote their health, safety and independence.^9^^,^ 10 In the US, 8 million workers identified as also providing care to an adult family member, often for an age-related condition.^10^ Analysis of data from 20 European countries reported that, on average, 34.3% of the population were providing informal care, with a further 7.6% regarded as “intensive carers” (providing at least 11 hours of care per week) to a family member or friend.^11^

With Brazil’s growing elderly population, set to become the 6^th^ largest in the world by 2025,^12^ the role of carers in supporting older people to live well is increasingly important. One study of community-dwelling older adults (n = 1593) reported that 10.6% were limited in their ability to perform basic activities such as bathing and dressing, while 34.2% needed assistance with instrumental activities including shopping and using the telephone.^13^ More recently, a study of 71 people aged over 60 and their family carers living in a lower socioeconomic area in Brazil found that just over 82% of the older people were dependent to some degree.^14^

When caring for an older person, family members may perform activities ranging from transportation, cooking and housework to helping the person with hygiene, mobility, medications and finances.^10^ Additionally, family carers can be instrumental in navigating fragmented care systems on behalf of older relatives,^15^ and see their role as organizing care and transferring information between stakeholders.^16^ This role becomes even more important as the global population ages and hospital lengths of stay shorten, requiring older patients and their carers to assume more of the “burden of treatment” in the community.^17^

The functional health literacy of carers of older people should be assessed, given their potential to influence health-related communication,^18^ and help the older person access and use information to manage their own health.^19^^,^ 20 A recent systematic review found that carers’ health literacy has implications for the care recipient and the wider community.^21^ Despite considerable variation in tools and cut-off criteria, up to half of included carers were reported to have inadequate health literacy levels. Moreover, lower health literacy scores in carers were associated with fewer self-management behaviours and increased health service use.^21^^,^ 22

In view of the many tasks for which carers become responsible, including assisting in activities of daily living, with medications, medical support and also health decision-making of the elder, it is vital that carers are prepared to identify health issues of the elder and know which resources to employ. When the skills needed are lacking, a number of consequences can arise in terms of health outcomes of older people.^23^

In 1995, the TOFHLA - Test of Functional Health Literacy in Adults - was developed to measure reading and understanding skills in situations commonly encountered in the health system. Test scores allow the assessment of reading skills by measuring understanding, including the ability to read and understand text and numerical information.^24^ In 1999, Baker et al. developed a short version of the test, the S-TOFHLA, containing two text passages and four numeracy passages, that has similar reliability and validity as the full version. The application time of the test was reduced from 22 minutes for the original version to 12 minutes for the short version.^25^ The S-TOFHLA has been widely used in older populations.^4^^,^ 26 The test has been translated and culturally adapted for use in Brazil.^27^

The objective of the present article was to assess the performance of carers of older people for functional health literacy using the S-TOFHLA and to identify carers’ characteristics associated with low functional health literacy.

## METHODS

This cross-sectional study was conducted at two public geriatric teaching clinics of the Botucatu School of Medicine (UNESP), Brazil, between 1st March 2015 and 31st December 2016.

The sample size was calculated based on a confidence interval of 95% and precision of 10% for the expected prevalence of functional illiteracy in the general population of 30%.^27^^,^ 28 A sample size of 80 individuals was thus determined. Inclusion criteria were carers of older people who performed one or more care tasks for the older people; attending the study clinics; aged ≥18 years; and able to understand the study rationale (based on subjective assessment by the researcher). If caregiver was aged ≥60 years then his/her cognitive status was assessed using the Mini-Mental State Exam (MMSE).^29^ The MMSE was applied only to carers aged ≥60 years given that cognitive impairments are more frequent from 60 years of age and older. Carers with lower-than-expected scores for their educational level were excluded from the study because cognitive impairment may be a confounding factor in performance on the S-TOFHLA.^29^ The cut-off scores for the MMSE, adjusted based on educational levels that constituted an exclusion for participation were: 18 (no formal education), 22 (1-4 years education), 24 (5-8 years), 26 (9-11 years) and 27 (≥12 years).^30^^,^ 31

Carers present in the clinic waiting room were randomly invited to take part. Data collection was performed by in-person interview at the doctor´s office, taking around 20 minutes (maximum 45 minutes), before or after the medical consultation of the older patient.

All participants were assessed using a questionnaire to collect sociodemographic data and information on the care given to the older individuals under their care, and by the S-TOFHLA.

The S-TOFHLA consists of two sections: the first for reading comprehension; and the second for numeracy comprehension. The reading comprehension test comprises two health-related text passages. The first text contains information on a gastrointestinal exam and the second contains rights and responsibilities of a patient admitted to a hospital for healthcare. Each passage has missing words and for each of these gaps the respondent must choose the word that best completes the phrase from four alternatives, filling a total of 36 gaps.^25^ The numeracy test comprises four items: two medication bottles containing instructions on taking the medicine, where the individual must understand the information on administration time of the medications and their interference with food; a card containing information on laboratory test results, from which carers must infer their results; and an appointment scheduling card for consultations in a hospital setting, whose data must be interpreted.^24^ The test is scored in two stages: the written stage, where each correctly filled gap is attributed 2 points; and the numeracy stage, with each test scoring 7 points. The caregiver´s healthcare literacy level is classified according to their performance on the test as inadequate (0-53 points), marginal (54-66) or adequate (67-100).^25^

Carers were screened for the presence of depression by the Patient Health Questionnaire (PHQ-2),^32^ because depression disorders can influence performance on the S-TOFHLA, particularly due to possible associated attention deficit.

All participants signed the Free and Informed Consent Form. The study was approved by the Research Ethics Committee of the Botucatu School of Medicine/UNESP (CAEE 40554515.7.0000.5411).

### Statistical analysis

The categorical variables were expressed as crude and relative values and associations of these variables among groups were determined using the Chi-squared test. Continuous variables were expressed as measures of central tendency: means and standard deviations (for normal distribution) or medians and interquartile ranges (for non-normal distribution). The multivariate analysis used the ordinal logistic regression model with S-TOFHLA scores as the dependent variable. All statistical analyses were performed using the applications SPSS v.22 and STATA v.14 (Data Analysis and Statistical Software). The level of statistical significance adopted was 0.05.

## RESULTS

Ninety-seven individuals were invited to take part, 17 of whom refused to participate in the study. Eighty carers were interviewed, mean age 54.6 (± 11.7) years; 87.5% female. Further sociodemographic data are given in Table 1.

**Table 1:** Sociodemographic data of caregivers of older people who completed the S-TOFHLA test.

		N (%)
Sex	Male	10 (12.5)
	Female	70 (87.5)
Race	White	65 (81.3)
	Mixed (Black/White)	13 (16.3)
	Black	2 (2.4)
Marital status	With companion	52 (65)
	Without companion	28 (35)
Education	1-4 years	19 (23.8)
	5-8 years	15 (18.8)
	9-11 years	17 (21.3)
	≥12 years	29 (36.1)

Ninety-five percent (76 individuals) of the interviewees had not done a course for carers of older people, whereas four (5%) had done a training course.

Regarding activities that can be associated with degree of functional literacy of the caregiver, 83.8% were responsible for purchasing medications, 73.8% for administering medications and 100% for accompanying at medical consultations (Table 2).

**Table 2:** Activities performed by caregivers of older people who completed the S-TOFHLA test.

Activities performed as caregiver*	N (%)
Personal hygiene	33 (41.3)
Purchasing medications	67 (83.8)
Administering medications	59 (73.8)
Accompanying at medical consultations	80 (100)
Preparing meals	55 (68.8)
House cleaning	52 (65)

* caregiver could rate any number of activities as being performed

Based on the S-TOFHLA test results, carers were classified as having adequate (72.5%), marginal (12.5%) or inadequate literacy (15%) (Table 3).

**Table 3:** Performance of caregivers of older people on S-TOFHLA test.

S-TOFHLA	N (%)
Adequate (0 to 53)	58 (72.5)
Marginal (54 to 66)	10 (12.5)
Inadequate (76 to 100)	12 (15)

The results for associations of S-TOFHLA score with clinical and sociodemographic data of carers are given in Table 4.

**Table 4:** Association of performance of caregivers of older people who completed the S-TOFHLA with sociodemographic and clinical data.

Variable		S-TOFHLA			#p
		Inadequate	Marginal	Adequate	
Sex	Male	2	2	6	0.6
	Female	10	8	52	
Race	White	8	8	49	0.4
	Black or brown	4	2	9	
Marital status	With companion	8	6	38	0.9
	Without companion	4	4	20	
Education	1-4 years	9	6	4	<0.001
	5-8 years	1	1	13	
	9-11 years	2	1	14	
	≥12 years	0	2	27	
Caregiver course	Yes	0	1	3	0.6
	No	12	9	55	
Activities performed as caregiver	Personal hygiene	5	6	22	0.4
	Purchasing medications	10	9	48	0.8
	Administering medications	7	9	43	0.2
	Preparing meals	8	8	39	0.7
	House cleaning	7	7	38	0.8
Clinic	HCI*	2	1	6	0.8
	HCS**	10	9	52	
^##^PHQ-2 (depression screen)	Screened positive	4	4	18	0.8
	Screened negative	8	6	40	

* HCI, Health Center I;

** HCS, Health Center School;

# Chi-square test;

## PHQ-2, Patient Health Questionnaire.

There was a statistically significant difference considering S-TOFHLA scores between the groups of educational levels, where the proportion of carers with higher educational level was greater in the group with adequate S-TOFHLA scores, while the proportion with low education was higher in the groups with inadequate and marginal S-TOFHLA scores.

The individuals who completed the MMSE had compatible scores with their educational level with none testing positive for cognitive impairment.

According to the ordinal logistic regression model for individuals of the same age group, the caregiver group with 5-8 years of education had an 18 times greater likelihood than those with 1-4 years of education of having adequate health literacy level (OR:18.0; CI_95%_: 3.0-107.0). The 9-11 years education group had an 11 times greater likelihood of having adequate health literacy (OR:11.0; CI_95%_ 2.0-57.0) whereas the ≥ 12 years education group had a 38 times greater likelihood (OR:38.0; CI_95%_: 6.4-228.0), as depicted in Table 5.

**Table 5:** Analysis of the maximum likelihood estimates obtained by adjusting the ordinal logistic regression model.

		Degrees of freedom	Estimate	Standard error	p-value	OR	CI
Age		1	0.0225	0.5839	0.4448	1.023	0.965-1.084
Education (years)	5-8	1	1.4416	10.0085	0.0016	17.872	2.995-106.648
	9-11	1	1.1835	7.6108	0.0058	10.665	1.984-57.312
	≥12	1	1.8207	15.9442	<0.0001	38.142	6.385-227.838

## DISCUSSION

This is the first study in Brazil assessing functional literacy in carers of older people. As expected for the Brazilian population, educational levels were heterogeneous and functional health illiteracy was associated with lower educational levels. Thus, the provision of care to older people can be negatively impacted by low literacy, decreasing the quality of the service provided. In addition, barriers in the health education process may be present and so health professionals must be especially alert to prevent potential errors and mistakes and to use a wide range of resources to achieve the desired goals.^23^ In the present study, 75% of carers were involved in purchasing and administering medications used by the older person in their care.

The 27% rate of functional health illiteracy found in this study is in line with the most complete database on health literacy for the American population, derived from the NAAL - National Assessment of Adult Literacy. The NAAL was conducted in 2003 employing a bespoke scale and showed that a third of America´s population exhibited functional health illiteracy, impacting the reading, understanding and application of health information.^33^^,^ 34 Comparing with the general adult population in Brazil, the present study results are consistent with those of a Brazilian study conducted in the city of São Paulo/Brazil among adult users of the Brazilian National Health System (SUS), which found that 32% of these users had inadequate or marginal health literacy scores, as measured by the S-TOFHLA.^27^

The results of the present study are also consistent with an American study,^35^ that determined health literacy levels using the S-TOFHLA in Hispanic elderly patients, their carers and its associations, identifying a rate of inadequate health literacy of around 31% among carers. Another American study that assessed rates of functional health literacy using the full version of the TOFHLA in paid carers found a rate of inadequate health illiteracy of 33%.^22^

Comparison of data on carers of older people versus carers of children revealed similar results. A study performed in an American pediatrics hospital assessed the association between literacy levels in carers of children and Emergency Department use, observing that the lower the literacy level, the higher the use of non-urgent services in the Emergency Department.^36^

In the present study, an association between S-TOFHLA test scores and educational levels was found in carers, where higher educational level was correlated with higher health literacy level. This association was previously reported in both the USA^33^^,^ 34 ^,^ 36 and Brazil.^27^^,^ 37

The World Report on Aging suggests that these results can be partially explained by the social status of the population with greater educational level, since this group tends to have higher income and better access to health services compared to low educated groups. Many studies attribute the economic advantage to a combination of economic implications and psychosocial influences related to healthy behaviors, nutritional status and the way in which individuals seek medical services.^38^^,^ 39

The Surgeon General’s Workshop on Health Literacy also highlighted that limited health literacy is not an individual deficit but a systematic problem,^40^ and that action was needed in many spheres to address this problem.^39^

Low literacy in carers of elderly can have a direct negative impact on the health outcomes of the older person they provide care for. With regard to medication use, this can affect various aspects such as reading and interpreting medical prescriptions, treatment adherence, recognizing adverse reactions or therapy failure and can increase treatment costs.^23^^,^ 41

A study assessing the relationship between literacy and understanding prescription medication labels revealed that patients with low and marginal literacy frequently misunderstood medication labels.^42^ Another study assessed the relationship between health literacy and medication adherence, concluding that being skilled at understanding basic information on prescription medications significantly impacts the ability to adhere to therapy regimens.^43^

A study assessing the impact of health literacy on aspects of medication non-adherence in adults with type 2 diabetes concluded that low health literacy levels are associated with patient self-reported difficulty remembering to take medications.^44^ According to another study, difficulty understanding medical prescriptions and low adherence to therapy, associated with low health literacy levels, contribute to higher medication costs.^45^

Shifting epidemiological and demographic profiles in Brazil have led to an increase in the number of medications used by older people. Treatments for common diseases in old age such as hypertension, diabetes, osteoporosis and arthritis often call for the use of three or more drugs, leading to polypharmacy. Knowledge and control of the many different processes involved in medication use, including purchase, administration, when to take and treatment adherence is vital, where higher levels of health literacy among older people and carers may improve execution of these processes.^22^

Instruments for detecting health literacy can be used by health professionals to help identify difficulties experienced by carers and older people, allowing for the devising of strategies to improve their understanding of health needs.^25^

Several limitations of the present study should be taken into account. This was a cross-sectional study, thereby precluding the establishment of probable causal relationships with functional literacy levels. A convenience sample was employed, with individuals selected based on their availability for the study at specialized centers for the older people, although the sample is consistent with characteristics of the wider caregiver population. No instruments were applied to assess the work load of carers or the levels of dependency of the older person being cared for and, therefore, the influence of these factors on caregiver health literacy could not be established.

In conclusion, we conclude that the results of the present study are consistent with the data in the literature, showing that around 1/3 of carers of the older people studied had marginal or inadequate levels of health literacy. An association between health literacy and educational levels was found, although no relationship of health literacy with caregiver age was evident.

The study highlighted the importance of prospective studies assessing the possible impacts of low health literacy of carers on the lives of older people, such as emergency service use, number of hospital admissions and health complications due to poor management of health instructions. Health professionals could assess health literacy among carers of older people and thereby enhance medical advices for these carers and guarantee better care for the elderly.

In addition, the study also reiterates the importance of devising strategies for increasing health literacy levels to improve the care provided by carers and their understanding of health information.
